# The Relation between Hypertension and the Kidney

**Published:** 1952-07

**Authors:** Harry Sosnowick

**Affiliations:** Lecturer in Physiology, University of Bristol


### THE RELATION BETWEEN HYPERTENSION AND THE KIDNEY

### A Review

BY

HARRY SOSNOWICK, B.SC., M.B., CH.B.
*Lecturer in Physiology, University of Bristol*

It is easily shown that a decrease in the calibre of blood-vessels is a far more important immediate cause of hypertension than are changes in cardiac output or in blood volume or viscosity. In younger hypertensives, at least, the fall in blood-pressure seen after drugs or sympathectomy, and results of the cold-pressor test, show that the narrowing is primarily due to vascular spasm. Any organic narrowing is very likely to be a secondary effect, and medial thickening of arteries is to be regarded as a compensatory work hypertrophy (Hueper, 1944).

### THE KIDNEY AS THE INITIATOR OF VASOSPASM

Although Bright had noted the " full hard pulse " of his patients, he had no means of measuring their blood-pressure. The first unequivocal demonstration of a renal extract with a pressor action was given in 1898 by Tigerstedt and von Bergmann, the active substance being named *renin.*

Little more was learnt until 1934, when Goldblatt's experiments stimulated widespread investigation. These experiments are now so well known that a detailed description is unnecessary. It was found that removal of one kidney in a dog, with interference of the blood supply to the other, was usually followed by hypertension within ten minutes to forty-eight hours. Denervation of the kidney did not affect the outcome, which was therefore not due to a nervous factor. Subsequent work, reviewed by Newton and Calma (1949), led to the generally accepted view that the ischaemic kidney formed an enzyme, renin, which reacted with one of the plasma globulins to produce a pressor substance, hypertensin, and this stimulated a generalized arteriolar constriction. It has, however, never been conclusively proved that experimental hypertension is in fact due to renin, or that this substance is more than an accidental finding.

Grollman (1949) removed both kidneys from dogs, and maintained life and good health for thirty to seventy days with an artificial kidney. The dogs developed hypertension, and as no renal tissue was present, Grollman concluded that the pressor substance was formed elsewhere. In other dogs, the kidneys were allowed to remain, with an intact blood supply, but were rendered functionless by bilateral ligation of the ureters. Hypertension did not develop, and Grollman took this to mean that kidneys normally form an antagonist to an extra-renal pressor material. But this work does not disprove the renin theory; pressor substances may be formed elsewhere as well as in the kidney, and the kidney may form also an antipressor compound. There is, in fact, fairly good evidence (Helmer *et al.*, 1942) that the kidney does form hypertensinase, which destroys hypertensin.

Renin, though detectable in the renal vein after inducing experimental renal ischaemia, is rarely found in the peripheral circulation, except during the first few days of an experiment. However, renin was found in the heart and liver of shocked dogs, and Braun-Menendez (1951) suggested that it may be absorbed by the blood-vessel walls, and form hypertensin *in situ*, thus accounting for its absence from the circulation.

From further studies, it was argued that renin, being an enzyme and therefore a protein, should act as an antigen, and stimulate antibody production. Wakerlin (1952) has obtained an antibody to hog renin, which prevents the pressor effects of both hog and dog renin, but does not neutralize human renin, or lower human blood-pressure. So once again, the experiments neither prove nor disprove the significance of renin.

Pickering (1945) found that in rabbits removal of the one remaining kidney a week after ischaemia had been induced in it, was followed by the disappearance of hypertension, but if removal was postponed for two months, the blood-pressure was not affected. From these experiments, and from others demonstrating the absence of renin after the first few days of hypertension, he concluded that two factors were concerned in producing experimental hypertension. One factor, perhaps renin, initiated the condition; the other, at present unknown, factor maintained it. In further support of this view, hypertension persisted in the rat after removal of the ischaemic kidney. In women with toxaemia of pregnancy, it was noted that blood-pressure returned to normal more slowly, or not at all, when toxaemia was of long duration.

### THE HEPATO-RENAL OR VDM-VEM MECHANISM

During the past two years, Shorr and his group (Shorr, 1951a) have discovered another humoral mechanism, independent of renin, which can account for both the onset and the maintenance of hypertension. It is well known that only a proportion of the total number of capillaries and metarterioles in any one area are open. Their number, and degree of dilatation, appear to be influenced by many chemical substances, some produced locally, and others at special sites. Among the latter are two important ones, a vasodilator material (VDM) formed in the liver, and a vasoconstrictor substance (vasoexcitor material or VEM) produced only by the kidney. If VDM is present in excess, more vessels dilate, and blood-pressure falls; with excess VEM, the reverse occurs. Either substance can be estimated by a bio-assay method.

The oxidized form of VDM, which is inactive so far as blood-vessels are concerned, is, oddly enough, the iron-carrying protein, ferritin. Most of the VDM produced by the liver is normally in the form of ferritin; only a small amount is secreted as the reduced active compound. Less is known about VEM; although it has protein properties, it appears to be distinct from renin. Ischaemia, such as follows the Goldblatt experiment, stimulates VEM secretion, so that the blood pressure rises. More VDM is then formed, and this is in the unoxidized active form. The balance between the amounts of VEM and VDM is such that the blood-pressure is stabilized at a higher level than before. Shorr (1951b) has reviewed many similarities between experimental renal hypertension in animals and the human disease. With tetrazolium, specific histo-chemical changes have been demonstrated in the proximal renal tubules of hypertensives for the first time, and these may be related to VEM secretion.

### THE INTRA-RENAL CIRCULATION

Trueta and his co-workers (1947) have suggested a possible mechanism for production of renal ischaemia in man. By injecting rabbit kidneys it was found that blood had two possible intrarenal routes, either (a) through glomeruli in the outer two-thirds of the cortex (cortical glomeruli), then to the capillaries around the whole length of the adjacent tubules, and finally to the veins, or (b) through the glomeruli in the inner third of the cortex (juxtamedullary glomeruli), then along vessels running with the U-shaped loops of Henle, and so to the veins. Similar results were found in many other species, including man.

Trueta makes a further claim, which will not be accepted, that either circulation may shut down independently of the other. Closing the cortical circulation would by-pass eighty-five per cent. of nephrons, an ample stimulus for renin or VEM release. Such an ischaemia is detectable by a pallor of the renal cortex, and can be obtained in the rabbit by such stimuli as a considerable haemorrhage or faradization of a cut sciatic nerve. But these are severe stimuli, not comparable with those presumed to initiate human hypertension, and the rabbit has a notoriously labile sympathetic system. Its kidneys differ from those of most mammals; Dicker and Heller (1945), for instance, found the rabbit glomeruli open and close, as do those of amphibia, whereas Smith (1943) showed that all glomeruli remain continuously open in the human kidney. Smith (1951) has very fully reviewed a mass of evidence, accumulated by many workers, and shown that the concept of a renal shunt is inconsistent with the findings in man during adult life and in senescence, after adrenaline or histamine, in syncope, shock, anuria and congestive heart failure, during diuresis, during abdominal compression, in pyrexial hyperaemia and finally in hypertension. As this evidence is readily available in Smith's review, there is no need to elaborate it here.

The direct evidence for a shunt in man comes from reports of patients with renal cortical necrosis (e.g. Jeffcoate, 1950), but even this evidence is disputed by some (Selkurt, 1951) who distinguish between cortical ischaemia and a true diversion of the cortical blood through the juxta-medullary circulation. Altogether it seems that the shunt is a mechanism found in rabbits but not in man.

Authors have at times claimed the existence of direct intrarenal arterio-venous anastomoses, thus providing another basis for renal ischaemia. In one recent paper (Trabucco and Marquez, 1952), it was even claimed that afferent and efferent glomerular vessels are continuous, and glomeruli are *cul-de-sacs* at the ends of side-branches. Other workers have injected glass spheres up to 0·4 mm. diameter into the renal artery, and recovered them from the renal vein; being too large to pass through the glomeruli, these spheres have presumably travelled by arterio-venous connections. But the most carefully made kidney injections, those of Trueta, did not show any such connections in normal kidneys, and those which are present appear to result from glomerular degeneration. Smith points out that the recovery of the glass spheres can be explained by the presence of a few anomalous vessels, such as may be expected among the million nephrons of each kidney.

### RENAL BLOOD-FLOW IN HUMAN BENIGN HYPERTENSION

Smithwick (1951) took biopsies from kidneys during sympathectomies performed for hypertension. In five hundred patients with long-standing hypertension, intrarenal vascular changes were absent in forty-five per cent.; in earlier cases, kidneys were more frequently normal. This suggests that if renal ischaemia is present in the human disease, it is, in fact, due to intrarenal vascular spasm, and not to renal arteriolosclerosis. Smirk (1949) in an important review on the pathogenesis of essential hypertension, concluded that when hypertension " is present in comparatively young people, the elevation of blood-pressure is often, probably usually, manifest before the characteristic pathological changes make their appearance in the kidney. . . ."

Smith *et al.* (Goldring, 1944) found by the usual clearance methods, that renal blood flow was essentially normal in the earlier cases of hypertension, although it was reduced when the disease was more advanced. They concluded that hypertension was the precursor of ischaemia, rather than *vice versa*. Chasis and Redish (1941) measured the blood flow of the two kidneys separately in each of several hypertensive patients, and found the two sides had an approximately equal flow. This finding is more likely if the hypertension precedes the ischaemia, rather than the reverse.

It may be concluded that evidence for renal ischaemia preceding benign hypertension in the human is scanty. Humoral theories, so far as the disease and not the experimental condition is concerned, receive little support from renal blood flow measurements.

### IS NEPHRECTOMY EVER INDICATED IN HYPERTENSION?

To summarize, it would seem on theoretical grounds that nephrectomy would have little importance in the treatment of hypertension, and this view is borne out by clinical experience. Rath and Russek (1945) found urological disease present in 10·7 per cent. of 6,044 hypertensive patients; even so, it does not necessarily follow that the former gives rise to the latter. The figure of ten per cent., however, seems an average one, and suggests that urological disease is not an important cause of hypertension. Nephrectomy, of course, can be of use only when unilateral renal disease gives rise to the hypertension, and this must occur quite rarely. Goldring and Chasis (1944) found hypertension no more commonly in those with such renal disease than in an unselected group of patients; and most other workers report similarly, although there are exceptions (Braasch, 1940; Baggenstoss, 1941). The acid test of the value of nephrectomy is to find how frequently it has in practice relieved hypertensive patients of their disability. Smith (1948) found 107 partial or complete cures of hypertension in 242 reported instances of unilateral nephrectomy. A few more successful cases have been reported since (e.g. Wayman and Ferris, 1952; Hoffman *et al.*, 1952), but the number is very small in relation to the total number of hypertensives. The urological condition most commonly found in successful cases is a unilateral atrophic pyelonephritis, or a congenitally small or kinked renal artery. Smith concludes such patients form 0·5 per cent. of all hypertensives. Until we have a reliable means of deciding beforehand which patients belong to this small group, nephrectomy can have no place as a planned treatment for hypertension.

### REFERENCES

Baggenstoss, A. H., and Barker, N. W. (1941). *Arch. Path.* **32**, 966.
Bell, E. T. (1951). *Hypertension*, Minneapolis: Univ. of Minnesota Press.
Braasch, W. F., Walters, W., and Hammer, H. J. (1940). *J. Amer. Med. Assoc.* **115**, 1837.
Braun-Menendez, E. (1951). In Bell, *supra*, p. 164.
Chasis, H., and Redish, J. (1941). *J. Clin. Investigation*, **20**, 655.
Dicker, S. E., and Heller, H. (1945). *J. Physiol.* **103**, 449.
Goldblatt, H. (1951). In Bell, *supra*, p. 5.
Goldring, W., and Chasis, H. (1944). *Hypertension and Hypertensive Disease*, p. 114. New York: Commonwealth Fund.
Grollman, A., Muirhead, E. E., and Vanatta, J. (1949). *Am. J. Physiol.* **157**, 21.
Helmer, O. M., Kohlstaedt, K. G., and Page, I. H. (1942). *Fed. Proc.* **1**, 114.
Hueper, W. C. (1944). *Arch. Path.* **38**, 245.
Jeffcoate, T. N. A. (1950). Quoted by Franklin, K. J., in *Proc. Roy. Soc. Med.* 1950, **43**, 475.
Newton, W. H., and Calma, I. (1949) in Newton, W. H., *Recent Advances in Physiology*, 7th ed. 1949, pp. 113–31. London: Churchill.
Pickering, G. W. (1945). *Clin. Sc.* **5**, 229.
Rath, M. M., and Russek, H. I. (1945). *Amer. Heart J.* **29**, 516.
Selkurt, E. E. (1951). *Ann. Rev. Physiol.* **13**, 242.
Shorr, E. (1951 *a*). In Bell, *supra*, pp. 79–93.
Shorr, E. (1951 *b*). Ibid. pp. 265–77.
Smirk, F. H. (1949). *Brit. Med. J.* **1**, 791.
Smith, H. W. (1943). *J. Mt. Sinai Hosp.* **10**, 59.
Smith, H. W. (1948). *Amer. J. Med.* **4**, 724.
Smith, H. W. (1951). *The Kidney*, pp. 702–45. New York: Oxford Univ. Press.
Smithwick, R. H. (1951). In Bell, *supra*, pp. 201 *et seq.*
Trabucco, A., and Marquez, F. (1952). *J. Urol.* **67**, 235.
Trueta, J., Barclay, A. E., Daniel, P. M., Franklin, K. J., and Prichard, M. L. (1947). *Studies on the Renal Circulation.* Oxford: Blackwell.
Wakerlin, G. E. (1952). *J. Urol.* **67**, 27.

